# Extrahepatic disease clusters and mortality in people with steatotic liver diseases: a prospective analysis of 64,749 females and 113,587 males in the UK Biobank

**DOI:** 10.1186/s12916-025-04288-4

**Published:** 2025-07-31

**Authors:** Qi Feng, Chioma N. Izzi-Engbeaya, Thomas Beaney, Alexander G. Smith, Pinelopi Manousou, Mark Woodward

**Affiliations:** 1 The George Institute for Global Health (UK), School of Public Health, Faculty of Medicine, Imperial College London, 58 Wood Lane, London, W12 7RZ UK; 2 Section of Investigative Medicine and Endocrinology, Department of Metabolism, Digestion and Reproduction, Faculty of Medicine, Imperial College London, London, UK; 3 Department of Endocrinology, St Mary’s Hospital, Imperial College Healthcare NHS Trust, London, UK; 4 School of Public Health, Faculty of Medicine, Imperial College London, London, UK; 5 Division of Digestive Diseases, Department of Metabolism, Digestion and Reproduction, Faculty of Medicine, Imperial College London, London, UK; 6 Department of Hepatology, St Mary’s Hospital, Imperial College Healthcare NHS Trust, London, UK; 7 The George Institute for Global Health (Australia), University of South New Wales, Sydney, Australia

**Keywords:** Steatotic liver disease, Multimorbidity, Disease cluster, Mortality, Latent class analysis, Sex difference

## Abstract

**Background:**

Steatotic liver disease (SLD) is the most prevalent chronic liver disease worldwide and linked to various liver and extrahepatic diseases. However, the clustering of extrahepatic conditions and their impact on mortality in individuals with SLD remain poorly understood.

**Methods:**

We used UK Biobank data to identify sex-specific disease clusters among individuals with SLD and multimorbidity (having ≥ 2 extrahepatic diseases) using latent class analysis. Multivariable Cox models were used to assess associations between multimorbidity, disease clusters and all-cause mortality and mortality from cardiovascular diseases (CVD), extrahepatic cancers, liver-related diseases and hepatocellular carcinoma.

**Results:**

Among 178,336 (36.3% female) individuals with SLD, during a median follow-up of 13.8 years, multimorbidity increased mortality by 100% (hazard ratio (95% confidence interval): 2.00 (1.93, 2.08)) and 80% (1.80 (1.71, 1.90)) in males and females, respectively, and increased the risk of death from CVD, extrahepatic cancers and liver-related diseases. Among 36,002 (43.9% female) of the 178,336 with multimorbidity, we identified five disease clusters in both sexes: related to respiratory, mental health, cancer/osteoarthritis and cardiovascular diseases. Males had separate heart and stroke clusters, whereas females had a combined heart/stroke cluster and a unique thyroid cluster. CVD was the leading cause of death in cardiovascular clusters, whereas extrahepatic cancers were the most common cause of death in other clusters. Among all disease clusters, cardiovascular clusters exhibited the highest all-cause mortality risk: 2.90 (2.64, 3.20) for the heart/stroke cluster in females and 2.63 (2.48, 2.78) for the heart cluster and 2.36 (2.16, 2.58) for the stroke cluster in males. All clusters exhibited increased mortality of CVD and extrahepatic cancers.

**Conclusions:**

Multimorbidity doubled the death rate in people with SLD. Common multimorbidity clusters of mental health, respiratory, cancer and cardiovascular diseases were found and were associated with varying mortality, with cardiovascular-related clusters showing the highest risk. Females exhibited a unique thyroid disease cluster. These findings highlight the need for tailored prevention and management strategies in SLD populations.

**Supplementary Information:**

The online version contains supplementary material available at 10.1186/s12916-025-04288-4.

## Background

Steatotic liver disease (SLD) is the most prevalent chronic liver disease, affecting every one in three adults worldwide [1]. SLD can progress to liver fibrosis, cirrhosis and hepatocellular carcinoma (HCC) and end-stage liver disease [2, 3]. SLD is closely linked to excess alcohol consumption and/or cardiometabolic risk factors, such as obesity, hypertension, diabetes and dyslipidaemia [4]. Beyond liver-related conditions, it is also associated with increased risk of cardiovascular diseases (CVD), extrahepatic cancers and chronic kidney disease [5–7].

Multimorbidity (MM), the coexistence of two or more long-term conditions (LTCs), is increasingly recognised as a major public health challenge, particularly in ageing populations [8, 9]. People with SLD frequently exhibit multimorbidity, largely driven by cardiometabolic and cancer comorbidities. However, beyond these well-established associations, the broader multimorbidity patterns in individuals with SLD remain poorly characterised. Understanding these different phenotypes in the form of disease clusters may help to improve risk stratification, understand disease mechanisms and optimise clinical management and healthcare services, thus improving individual long-term outcomes [10].

Previous studies have used various clustering techniques to derive disease clusters in general populations, often identifying cardiometabolic, respiratory and mental health clusters [11, 12]. However, few studies have specifically examined extrahepatic multimorbidity clusters in individuals with SLD. Given the high burden of liver-related and extrahepatic complications in this population, it is essential to determine whether distinct disease clusters exist and how they may impact mortality outcomes. Additionally, sex differences in disease clustering remain under-explored [8].

This study aimed to identify multimorbidity clusters among individuals with SLD in the UK Biobank and to examine the associations between these clusters and all-cause and cause-specific mortality, considering sex differences in disease clustering and outcomes.

## Methods

### Data and participants

We used data from the UK Biobank, a prospective cohort of half million individuals aged 40–70 years old recruited between 2006 and 2010. Baseline assessment collected data on socioeconomic status, lifestyle factors, health status, occupational and environmental exposures and anthropometric measures. Biological samples of blood, urine and saliva were collected. All participants were followed up via linkage to national death registries and hospital records.

**Fig. 1:**
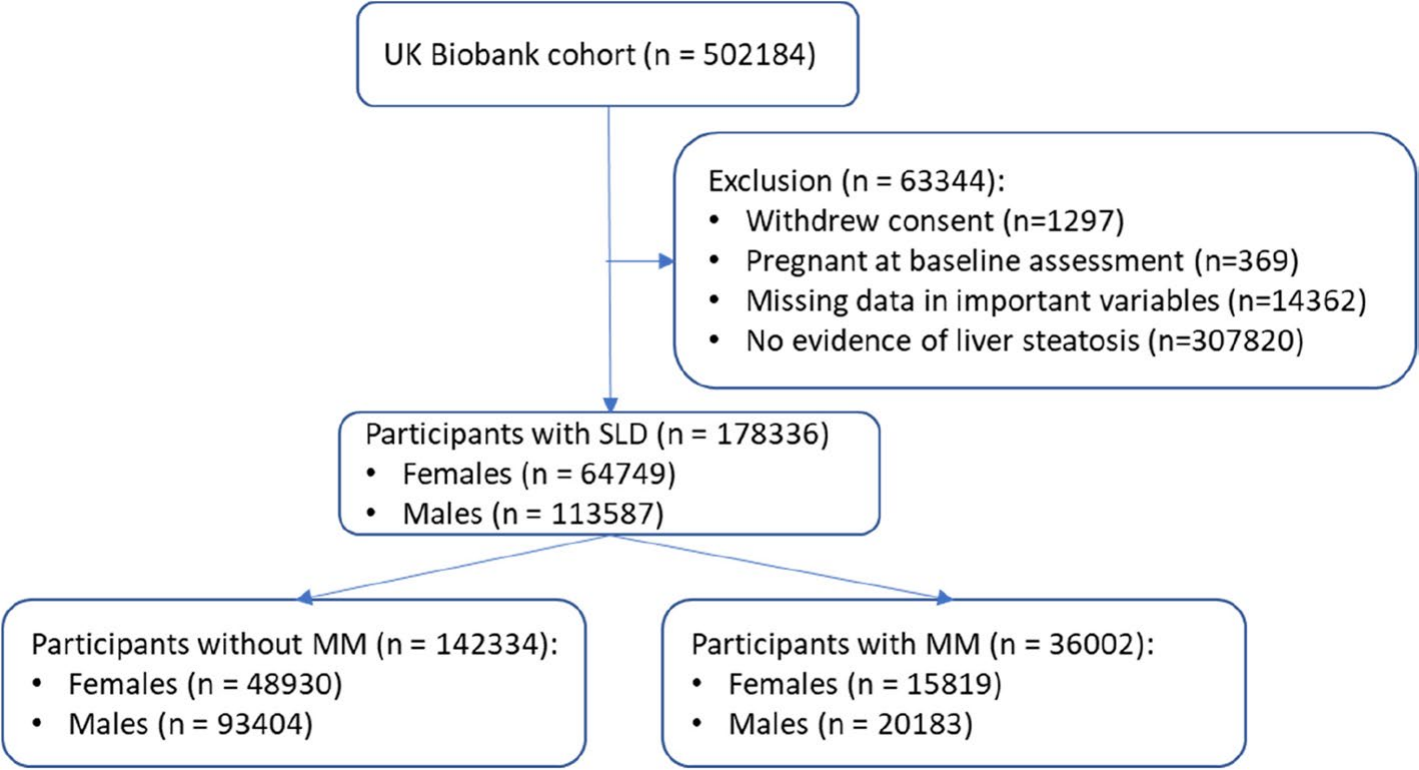
Flowchart of participant selection. SLD, steatotic liver disease; MM, multimorbidity. Note: some people were excluded for more than a single reason

We included people with SLD, defined using a fatty liver index (FLI) > 60 as an indicator of liver steatosis. FLI is a biomarker-based score for hepatic steatosis levels, calculated with body mass index (BMI), waist circumference, triglycerides (TG) and gamma-glutamyl transferase (GGT) [13]. The cutoff value of 60% has been validated and used previously to define liver steatosis [14, 15]. SLD subtypes were classified into metabolic dysfunction-associated steatotic liver disease (MASLD), metabolic dysfunction and alcohol related liver disease (MetALD) and alcohol related liver disease (ALD), based on alcohol consumption and the presence of cardiometabolic risk factors (CMRF) [3, 16]. CMRFs included obesity, hypertension, diabetes, high TG and low high-density lipoprotein (HDL) cholesterol, and we measured these CMRFs as defined in Rinella et al. [16]. We excluded people who withdrew from the cohort, who were pregnant at baseline and who had missing data in calculating FLI (Fig. 1).

### Multimorbidity and disease clusters

We defined multimorbidity (MM) as presence of at least two of 47 LTCs from a prespecified list. This list of disease was based on a three-round Delphi study of healthcare professionals and public members [17]. The criteria for including these LTCs included their impacts on mortality, quality of life, frailty, physical disability, mental health and treatment burden. Among the conditions identified in this Delphi study, we combined solid organ cancers, metastatic cancers, melanoma and treated cancer requiring surveillance, into one broad solid organ cancer category, and removed post-acute COVID-19, chronic Lyme disease and two liver conditions (HCC and chronic liver disease). Since SLD is our index disease, we excluded SLD and its associated CMRFs from the list, namely obesity and diabetes. After these modifications, this study considered 47 LTCs for multimorbidity (Additional file 1: supplementary methods). These LTCs covered extrahepatic cancers, cardiovascular, metabolic, endocrinological, respiratory, digestive, renal, mental/behavioural and congenital conditions. Diagnosis of these conditions in each individual was confirmed in self-report medical conditions at baseline assessment and hospital records on and before the date of attending the baseline assessment. The codelists for these LTCs are presented in Additional file 1: supplementary methods.

We used latent class analysis (LCA) [18] to determine disease clusters in a binary disease presence matrix in individuals with multimorbidity (≥ 2 LTCs), separately in females and males. LCA allocates each individual to a single non-overlapping cluster and allows each condition to contribute to various clusters by varying probabilities. The algorithm produced posterior probabilities of cluster membership for each individual, and each individual was assigned to the cluster for which they have the highest posterior probability. In this way, each individual was assigned to one and only one cluster; thus, the clusters were mutually exclusive. We compared the performance statistics for multiple LCA models of between 2 and 12 clusters, using the Bayesian information criterion (BIC), sample size-adjusted BIC (aBIC) and Akaike information criterion (AIC). The optimal number of clusters was decided based on three criteria: (1) a lower aBIC value (indicating better model fitting); (2) the minimal size of any cluster should be ≥ 5% of the total sample for LCA, to avoid overly small clusters, which may indicate overfitting; and (3) clinical interpretability of the resulting clusters, via consultation with clinician coauthors (PM, CI and TB). These criteria have been used in previous publications [11, 12].

The derived clusters were characterised by the 3 LTCs with the highest probabilities ≥ 5% of contributing to that cluster, excluding conditions for which their prevalence in the cluster was equal to or less than their prevalence in the total sample [11]. Additionally, we calculated observed/expected prevalence ratio (O/E ratio) and exclusivity for each characteristic LTC, as in Roso-Llorach et al.’s study [19]. O/E ratio was calculated by dividing the prevalence of a given LTC within a cluster by its prevalence in the overall sample for LCA. LTC exclusivity was defined as the fraction of participants with the LTC in the cluster over the total number of participants with the LTC. LTCs with exclusivity ≥ 20% or O/E ratio ≥ 2 were considered strong characteristic diseases for the clusters. This approach ensured that only relatively common and cluster-distinctive diseases were used to define the cluster profiles.

### Outcome and covariates

The outcomes were all-cause mortality and mortality of HCC, liver-related diseases, CVD and extrahepatic cancers. These cause-specific mortality outcomes were chosen because they were the most common and clinically relevant causes of death in people with SLD [20, 21]. Cause of death was confirmed in death registry records. Participants were censored at the date of death or the last day of follow-up (30 November 2022), whichever occurred earlier. We used the following ICD10 codes for specific causes of death: HCC (C22.0), liver-related diseases (K70–K77, C22), CVD (I00–I99) and extrahepatic cancer (C00–C99 excluding C22). Liver-related diseases included chronic liver disease (K70-K77) and liver cancer (C22). The codelists have been used in previous studies [22] and reviewed by clinician coauthors (PM and CI) to ensure accuracy.

A participant’s region was determined by the location of the assessment centre they attended. Ethnicity was classified into White, Asian, Black and mixed/others. The Townsend deprivation index is a postcode-derived measure for socioeconomic status. Educational attainment was categorised as below secondary, lower secondary, upper secondary, vocational training and higher education. Lifestyle factors included self-reported current smoking (current, previous and never smoker), alcohol consumption and physical activity level. Alcohol consumption was assessed via self-reported weekly or monthly intake of various alcoholic drinks; the consumption was summed up to derive average daily alcohol consumption (g/day) [23]. Physical activity level was measured with the International Physical Activity Questionnaire; individuals were categorised into low, moderate and high levels, based on the frequency, duration and intensity of their physical activities. Systolic and diastolic blood pressure were measured twice and the averages of the two readings were used in analyses. Blood biochemistry markers were measured at a central laboratory, including TG, HDL cholesterol, HbA1c, ALT, AST and GGT. For all the categorical covariates, answers of “unknown”, “do not know” and “prefer not to say” were combined into one “unknown” category.

### Statistical analysis

The baseline characteristics of males and females with SLD were summarised and compared between those with multimorbidity and those without, as well as across the derived disease clusters.

Cox proportional hazards models were fitted to assess the associations between multimorbidity, disease clusters and mortality outcomes, expressed as hazard ratio (HR) and 95% confidence interval (CI), using individuals without multimorbidity as the reference group. Date of baseline assessment was used as the time origin. The models were stratified by region (England, Scotland, Wales) and age groups (< 50, 50–60, ≥ 60) and adjusted for ethnicity (White, Asian, Black, others), Townsend deprivation index (in equal fifths), education (below secondary, lower secondary, upper secondary, vocational training, higher education), smoking (current, previous, never), physical activity (low, intermediate, high) and alcohol consumption (continuous, g/day). The proportional hazards assumption was examined by scaled Schoenfeld residuals, and no evidence was observed for its violation. For sensitivity analyses, we (1) further adjusted for the five CMRFs and (2) removed the first 2 years of follow-up to correct for reverse causation.

### Cluster validation

We assessed the validity of the derived clusters, using three commonly recommended approaches: associations with clinical outcomes, clinical plausibility and stability across subsamples, as summarised by Dhafari et al. [24]. These aspects were incorporated into the study design and analyses. First, we evaluated clinical relevance by examining associations between the identified clusters and mortality outcomes. Second, clinical plausibility was assessed through consultations with clinician coauthors (PM, CI, TB), who reviewed the disease profiles of each cluster.

Third, to examine the stability of clustering process across subsamples, we conducted sensitivity analyses by randomly selecting 80% and 50% of the full sample and repeating LCA. Across the three samples (i.e. full, 80% and 50% samples), we compared the results with respect to the aBIC plot, the optimal number of clusters, the characteristic diseases for each derived cluster and their prevalences, the distribution of posterior probability and the proportion of individuals assigned to the same cluster.

Since individuals were assigned to clusters based on their highest posterior probability, individuals with higher posterior probability were assigned to a cluster with more confidence than those with lower probability. To examine the impact of potential uncertainty in cluster assignment, we conducted a sensitivity analysis excluding the participants with a maximum posterior probability below 70% (approximately the lowest quartile) and estimated the associations between the disease clusters and mortality outcomes in the remaining participants, to examine the robustness of cluster assignment.

All analyses were done in R.

### Ethics approval and consent to participate

UK Biobank received ethical approval from the National Information Governance Board for Health and Social Care and the National Health Service North West Multi-centre Centre for Research Ethics Committee (Ref: 21/NW/0157). All participants provided informed consent at recruitment to the study for their data to be used for health-related research that was in the public interest.

UK Biobank has approval from the North West Multi-centre Research Ethics Committee as a Research Tissue Bank approval. This approval means that researchers do not require separate ethical clearance.

## Results

We included 178,336 individuals with SLD in analysis (36.3% females, mean age 57.3 years; 73.5% MASLD, 19.0% MetALD, 6.4% ALD) (Fig. 1). About one in five (20.2%) of SLD participants had MM, higher in females than in males (24.4% vs. 17.8%). The prevalence of MM was 21.3%, 15.9% and 17.4% in people with MASLD, MetALD and ALD, respectively. Overall, compared to people without MM, people with MM were more likely to be older, living in socioeconomically deprived area, less educated, smokers and drink less. They were also more likely to have diabetes and low HDL cholesterol, but less likely to have hypertension and high TG (Table 1).

**Table 1:** Baseline characteristics in participants with steatotic liver disease stratified by sex and multimorbidity status

	Males		Females		Overall
	MM	No MM	MM	No MM	
	*n* = 20,183	*n* = 93,404	*n* = 15,819	*n* = 48,930	*n* = 178,336
**Age, years**	59.4 (7.5)	56.5 (8.0)	58.6 (7.4)	57.4 (7.5)	57.3 (7.8)
**Townsend deprivation index**					
1st fifth (least deprived)	3188 (15.8%)	18,637 (20.0%)	2188 (13.8%)	8449 (17.3%)	32,462 (18.2%)
5th fifth (most deprived)	5728 (28.4%)	18,804 (20.1%)	4853 (30.7%)	11,485 (23.5%)	40,870 (22.9%)
Higher level of education	4727 (23.4%)	27,880 (29.8%)	3157 (20.0%)	11,582 (23.7%)	47,346 (26.5%)
White ethnicity	19,263 (95.4%)	88,343 (94.6%)	14,853 (93.9%)	45,724 (93.4%)	168,183 (94.3%)
Never smoked	7254 (35.9%)	42,439 (45.4%)	7983 (50.5%)	27,847 (56.9%)	85,523 (48%)
Alcohol drinking, g/day*	15.1 (0.0, 33.3)	19.5 (5.9, 36.7)	0.8 (0.0, 10.3)	3.4 (0.0, 13.1)	11.9 (0.0, 28.6)
High level of physical activity	5142 (25.5%)	29,056 (31.1%)	3042 (19.2%)	10,595 (21.7%)	47,835 (26.8%)
BMI, kg/m^2^	31.1 (4.4)	30.3 (3.7)	33.9 (5.3)	33.2 (4.7)	31.5 (4.5)
FIB4 score*	1.4 (1.1, 1.8)	1.2 (1.0, 1.6)	1.1 (0.9, 1.4)	1.1 (0.9, 1.4)	1.2 (0.9, 1.5)
Waist circumference, cm	106.4 (10.8)	103.4 (9.5)	101.7 (10.8)	99.6 (9.7)	102.6 (10.0)
Systolic blood pressure, mmHg	141.3 (17.6)	143.9 (17.0)	139.6 (18.0)	141.5 (18.3)	142.6 (17.6)
Diastolic blood pressure, mmHg	83.9 (10.3)	86.7 (9.7)	83.6 (9.9)	85.2 (9.5)	85.7 (9.8)
ALT, log_10_ U/L	1.4 (0.2)	1.5 (0.2)	1.4 (0.2)	1.4 (0.2)	1.4 (0.2)
AST, log_10_ U/L	1.4 (0.2)	1.4 (0.1)	1.4 (0.2)	1.4 (0.1)	1.4 (0.2)
GGT, log_10_ U/L	1.7 (0.3)	1.7 (0.3)	1.6 (0.3)	1.6 (0.3)	1.6 (0.3)
Triglycerides, mmol/L	2.1 [1.4]	2.2 [1.4]	2.0 [1.2]	2.0 [1.2]	2.1 [1.3]
HDL cholesterol, mmol/L	1.1 (0.4)	1.1 (0.4)	1.3 (0.4)	1.3 (0.4)	1.2 (0.4)
HbA1c, mmol/mol	38.5 (11.6)	36.2 (9.3)	38.1 (10.6)	36.8 (9.3)	36.8 (9.8)
Platelet, 10^9^ cells/L	237.7 (60.9)	238.7 (54.9)	277.1 (66.3)	274.7 (61.4)	251.8 (61.1)
Hypertension	15,450 (76.5%)	77,040 (82.5%)	13,038 (82.4%)	39,889 (81.5%)	145,417 (81.5%)
Obesity	19,788 (98.0%)	91,381 (97.8%)	15,788 (99.8%)	48,837 (99.8%)	175,794 (98.6%)
Diabetes	7795 (38.6%)	23,050 (24.7%)	6182 (39.1%)	14,735 (30.1%)	51,762 (29.0%)
High triglycerides	13,759 (68.2%)	67,561 (72.3%)	12,287 (77.7%)	34,685 (70.9%)	128,292 (71.9%)
Low HDL cholesterol	7266 (36.0%)	27,621 (29.6%)	10,506 (66.4%)	27,784 (56.8%)	73,177 (41.0%)

Values shown are *n* (%) except where units of measurement are stated where mean (standard deviation) is shown, except * showing median (interquartile interval)

*MM* Multimorbidity, *BMI* Body mass index, *FIB4* Fibrosis-4 score, *ALT* Alanine aminotransferase, *AST* Aspartate aminotransferase, *GGT* Gamma-glutamyl transferase, *HDL* High-density lipoprotein, *HbA1c* Glycated haemoglobin

### Disease clusters and their characteristics

Latent class analysis derived five distinct disease clusters for males and females, respectively, with some overlapping and some sex-specific patterns (Additional file 1: Fig. S1(A), Table S1). For males, although the clustering evaluation metrics indicated that an 8-cluster solution was statistically optimal, we opted against this model due to concerns on clinical redundancy and potential overfitting. Upon review, the 8-cluster solution included two highly similar respiratory clusters (one characterised by asthma and COPD, the other by asthma and other chronic respiratory disease), as well as two overlapping cardiovascular clusters (one with ischaemic heart disease, heart failure and arrhythmia, and another with ischaemic heart disease, heart failure and heart valve disease). A similar issue of cluster overlap was also observed in the 6-cluster solution. Therefore, to balance clinical interpretability and parsimony, we selected the 5-cluster model for subsequent analyses.

Table 2 summarises the characteristic diseases within each cluster. In both sexes, a respiratory cluster (characterised by asthma, COPD and other chronic respiratory diseases), a mental health cluster (dominated by depression and anxiety, with substance use disorder additionally present in males) and a cancer/osteoarthritis cluster (including solid organ cancers, chronic respiratory diseases and osteoarthritis) were observed. Among males, a heart cluster (characterised by ischaemic heart disease, arrhythmia and heart failure) and a stroke cluster (characterised by stroke and paralysis) were identified. In females, these two clusters merged into a single heart/stroke cluster, which included ischaemic heart disease, arrhythmia and stroke. Additionally, females had a unique thyroid cluster, consisting of thyroid disorders and connective tissue diseases. All these characteristic diseases showed high exclusivity (> 20%) and most showed high E/O ratio (> 2.0). Additional file 1: Table S2 shows more details on the LTCs in each cluster.

**Table 2:** Latent class analysis derived disease clusters in males and females with steatotic liver disease

	Males	Females
Cluster name	Respiratory cluster (*n* = 5587, 27.7%)	Respiratory cluster (*n* = 4116, 26.0%)
Characteristic diseases	o Asthma (100%) o COPD (16%) o Other chronic respiratory diseases (43%)	o Asthma (100%) o COPD (17%) o Other chronic respiratory diseases (40%)
Cluster name	Mental health cluster (*n* = 2814, 13.9%)	Mental health cluster (*n* = 3469, 21.9%)
Characteristic diseases	o Depression (80%) o Anxiety (28%) o Substance use disorder (19%)	o Depression (100%) o Anxiety (14%)
Cluster name	Cancer/osteoarthritis cluster (*n* = 6060, 30.0%)	Cancer/osteoarthritis cluster (*n* = 3396, 21.5%)
Characteristic diseases	o Solid organ cancers (29%) o Other chronic respiratory diseases (41%) o Osteoarthritis (24%)	o Solid organ cancers (31%) o Other chronic respiratory disease (35%) o Osteoarthritis (24%)
Cluster name	Stroke cluster (*n* = 1632, 8.1%)	Heart/stroke cluster (*n* = 1745, 11.0%)
Characteristic diseases	o Stroke (98%) o Paralysis (15%)	o Ischaemic heart disease (63%) o Arrythmia (34%) o Stroke (33%)
Cluster name	Heart cluster (*n* = 4090, 20.3%)	Thyroid cluster (*n* = 3093, 19.6%)
Characteristic diseases	o Ischaemic heart disease (83%) o Arrythmia (53%) o Heart failure (25%)	o Thyroid disorder (100%) o Connective tissue diseases (8%)

Showing the characteristic diseases and their prevalences in that cluster

*COPD* Chronic obstructive pulmonary disease

In males, the most common disease cluster to which individuals were assigned was the cancer/osteoarthritis cluster (30.0%), followed by respiratory cluster (27.7%), heart cluster (20.3%) and mental cluster (13.9%) while stroke cluster (8.1%) was the least common. In females, the most common cluster was respiratory cluster (26.0%), followed by mental cluster (21.9%), cancer/osteoarthritis cluster (21.5%) and thyroid cluster (19.6%), and the least common was heart/stroke cluster (11.0%) (Table 2). Across SLD subtypes, mental health cluster was more prevalent in ALD in both males and females, while heart/stroke and thyroid clusters were more prevalent in MASLD in females (Additional file 1: Fig. S2).

Additional file 1: Tables S3 and S4 present the baseline characteristics of males and females assigned to each disease cluster. In both males and females, clustering was associated with age, socioeconomic status, physical activity and CMRFs. Individuals in the respiratory and mental health clusters were younger, while those in the heart and/or stroke cluster were the oldest. Both sex-specific mental health and stroke clusters were more socioeconomically deprived, and the stroke clusters had lower level of physical activity. Both the stroke and heart clusters had higher prevalence of diabetes and low HDL than overall males. The heart cluster and stroke cluster also demonstrated differences in socioeconomic deprivation, physical activity level, diabetes, hypertension and low HDL cholesterol.

In females, the mental health and heart/stroke clusters were more likely to live in socioeconomically deprived area, with the heart/stroke cluster also less likely to have higher education, be never smokers or engage in high physical activity level. While diabetes was less common in the mental health cluster, it was more prevalent in the heart/stroke cluster, which also had higher TG and lower HDL levels. Mental health cluster was also more likely to be White ethnicity in females.

### Disease clusters and all-cause mortality

During a median follow-up of 13.8 years, 14,595 (12.8%) and 6171 (9.5%) deaths were captured in males and females, respectively. Compared to males with SLD but without MM, males with MM had an excess mortality rate of 12.3/1000 person-years (20.0 vs. 7.7), yielding an HR estimate of 2.00 (95%CI 1.93, 2.08). Compared to females without MM, females with MM had an excess mortality rate of 6.0/1000 person-years (11.7 vs. 5.7), responding to an HR estimate of 1.80 (95%CI 1.71, 1.90).

**Fig. 2:**
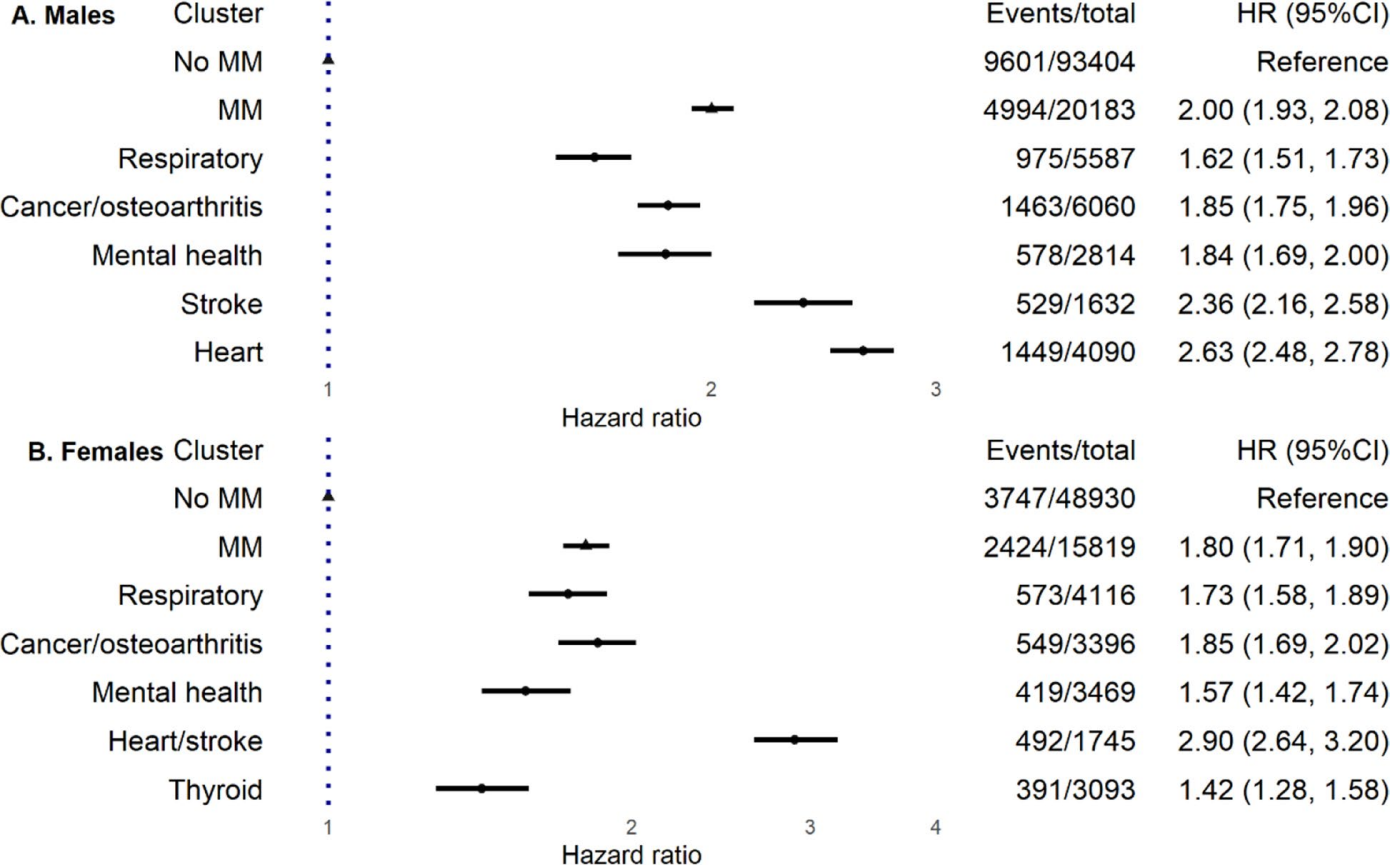
Associations between multimorbidity, disease clusters and all-cause mortality in males and females with steatotic liver disease. Model was stratified by region and age groups, adjusted for ethnic, education, deprivation, physical activity, alcohol intake and smoking. MM, multimorbidity; HR (95%CI), hazard ratio (95% confidence interval)

People in all disease clusters showed higher all-cause mortality than people without MM. In males, heart and stroke clusters were associated with the highest mortality, with HR of 2.63 (2.48, 2.78) and 2.36 (2.16, 2.58); respiratory, mental health and cancer/osteoarthritis clusters showed HR of 1.62 (1.51, 1.73), 1.84 (1.69, 2.00) and 1.85 (1.75, 1.96), respectively. In females, heart/stroke cluster was associated with the highest mortality, with HR of 2.90 (2.64, 3.20). Respiratory, mental health, cancer/osteoarthritis and thyroid clusters showed HR of 1.73 (1.58, 1.89), 1.57 (1.42, 1.74), 1.85 (1.69, 2.02) and 1.42 (1.28, 1.58), respectively (Fig. 2, Additional file 1: Table S5). Sensitivity analyses by additional adjustment for CMRFs and removing the first 2 years of follow-up generated similar results (Additional file 1: Table S6).

### Disease clusters and cause-specific mortality

Examining causes of death, 65% of all deaths were due to extrahepatic cancers, CVD and liver-related diseases, and the remaining 35% were due to other causes. More specifically, in males, 31.9% and 31.8% deaths were attributed to extrahepatic cancers and CVD, while only 1.1% to HCC and 4.4% to liver-related diseases. Similarly in females, 39.9%, 21.9%, 0.3% and 3.3% deaths were attributed to extrahepatic cancers, CVD, HCC and liver-related diseases, respectively. Extrahepatic cancer and CVD were the major causes of death across all clusters, contributing 58.1% to 67.3% of all deaths in males and 59.1% to 66.7% in females. CVD was the biggest cause of death in the stroke and heart clusters in males and in the stroke/heart cluster in females, while extrahepatic cancers remained the biggest cause of death for all other clusters (Additional file 1: Fig. S3).

Compared to people without MM, having MM was associated with higher mortality from extrahepatic cancers by 45% (1.45 (1.37, 1.54)), CVD by 173% (2.73 (2.56, 2.92)), HCC by 67% (1.67 (1.20, 2.32)) and liver-related diseases by 84% (1.84 (1.55, 2.18)) in males and extrahepatic cancers by 40% (1.40 (1.30, 1.52)), CVD by 130% (2.30 (2.04, 2.58)) and liver-related death by 46% (1.41 (1.06, 1.86)) in females. All five clusters were associated with increased mortality of extrahepatic cancers, with the highest risk in cancer clusters in males (1.77 (1.62, 1.92)) and females (1.74 (1.53, 1.98)). All five clusters were associated with mortality from CVD, with the highest risk in stroke and heart clusters in males (3.34 (2.86, 3.90) and 5.28 (4.83, 5.77)) and stroke/heart cluster (5.66 (4.75, 6.75)) in females. The disease clusters also showed general trend of positive associations with mortality of HCC and liver-related diseases, although non-significantly, likely due to the small number of events in these clusters (Table 3).

**Table 3:** Associations between the disease clusters and mortality of cardiovascular diseases and extrahepatic cancers, hepatocellular carcinoma and liver-related diseases in males and females with SLD

Clusters	Extrahepatic cancers		Cardiovascular disease		HCC		Liver-related diseases	
	Events/total	HR (95%CI)	Events/total	HR (95%CI)	Events/total	HR (95%CI)	Events/total	HR (95%CI)
Males								
No MM	4297/93,404	Reference	2241/93,404	Reference	119/93,404	Reference	439/93,404	Reference
MM	1595/20,183	1.45 (1.37, 1.54)	1590/20,183	2.73 (2.56, 2.92)	56/20,183	1.67 (1.20, 2.32)	208/20,183	1.84 (1.55, 2.18)
Respiratory	331/5587	1.24 (1.11, 1.39)	254/5587	1.80 (1.58, 2.05)	11/5587	1.48 (0.80, 2.76)	45/5587	1.65 (1.22, 2.25)
Mental health	177/2814	1.30 (1.12, 1.51)	159/2814	2.14 (1.82, 2.52)	8/2814	1.85 (0.89, 3.83)	39/2814	2.24 (1.60, 3.14)
Cancer/osteoarthritis	618/6060	1.77 (1.62, 1.92)	332/6060	1.83 (1.63, 2.06)	14/6060	1.30 (0.74, 2.27)	59/6060	1.69 (1.28, 2.22)
Stroke	163/1632	1.65 (1.41, 1.94)	175/1632	3.34 (2.86, 3.90)	4/1632	1.30 (0.47, 3.56)	13/1632	1.32 (0.76, 2.31)
Heart	306/4090	1.25 (1.11, 1.41)	670/4090	5.28 (4.83, 5.77)	19/4090	2.44 (1.49, 4.02)	52/4090	2.20 (1.64, 2.96)
Females								
No MM	1961/48,930	Reference	634/48,930	Reference	17/48,930	Reference	153/48,930	Reference
MM	966/15,819	1.40 (1.30, 1.52)	532/15,819	2.30 (2.04, 2.58)	8/15,819	1.02 (0.43, 2.43)	77/15,819	1.41 (1.06, 1.86)
Respiratory	228/4116	1.34 (1.17, 1.54)	113/4116	1.97 (1.61, 2.41)	4/4116	2.19 (0.72, 6.67)	14/4116	1.04 (0.60, 1.80)
Mental health	175/3469	1.26 (1.07, 1.47)	76/3469	1.70 (1.34, 2.16)	0/3469	NA	18/3469	1.54 (0.94, 2.52)
Cancer/osteoarthritis	265/3396	1.74 (1.53, 1.98)	101/3396	1.98 (1.60, 2.44)	1/3396	0.61 (0.08, 4.63)	18/3396	1.50 (0.92, 2.46)
Heart/stroke	141/1745	1.65 (1.38, 1.96)	168/1745	5.66 (4.75, 6.75)	1/1745	0.98 (0.12, 7.66)	13/1745	2.01 (1.13, 3.57)
Thyroid	157/3093	1.11 (0.95, 1.31)	74/3093	1.59 (1.25, 2.02)	2/3093	1.26 (0.29, 5.51)	14/3093	1.27 (0.73, 2.20)

Model was stratified by region and age groups, adjusted for ethnicity, education, Townsend deprivation index, physical activity, alcohol intake and smoking

*HCC* Hepatocellular carcinoma, *NA* Not applicable, *HR (95%CI)* Hazard ratio (95% confidence interval)

Stroke cluster males also had a lower mortality rate (26.9 vs. 30.1 per 1000 person-year) than heart cluster (Additional file 1: Table S5), with lower mortality of CVD but higher mortality of cancer compared to the heart cluster (Additional file 1: Fig. S3).

### Cluster validation

To assess the clustering stability across subsamples, we applied LCA to randomly selected 80% and 50% subsets of the full sample and compared the results with the primary full-sample analysis. We observed that the aBIC plots demonstrated similar trends across all samples, and the optimal number of clusters remains unchanged at 5 when applying all three model selection criteria (Additional file 1: Fig. S1(B, C)). The disease profiles of the derived clusters were highly consistent with those identified in the full-sample analysis (Additional file 1: Table S7). The distributions of posterior probability for each cluster were also similar across the three sample analyses (Additional file 1: Table S8). Comparing the full and 80% sample analyses, 99.0% males and 98.2% females were assigned to the same clusters; compared the full and 50% sample analyses, 96.3% males and 96.2% females remained in the same clusters. These results supported the robustness and reproducibility of the clustering solution.

To evaluate the impact of individuals with low posterior probability on the association estimates, we conducted a sensitivity analysis excluding participants with a maximum posterior probability < 70% for cluster assignment. This resulted in the exclusion of 5995 (29.7%) males and 3511 (22.2%) females (Additional file 1: Table S9). Additional file 1: Table S10 shows the associations between disease clusters and all-cause and cause-specific mortality outcomes, which were largely consistent with those observed in the primary analysis, further supporting the stability of clustering assignments.

## Discussion

In this prospective cohort including 36,002 participants with SLD and extrahepatic multimorbidity, we identified distinct disease clusters that differed by sex and were associated with varying mortality risks. While both sexes shared the respiratory, mental health, cancer/osteoarthritis and cardiovascular clusters, females had a unique thyroid cluster. Extrahepatic cancers emerged as the leading cause of death across most clusters, except for the cardiovascular clusters, where CVD predominated. MM doubled overall mortality, with the cardiovascular clusters exhibiting the highest mortality. MM was also associated with increased mortality from extrahepatic cancers, CVD and liver-related diseases, although the strength of these associations varied across clusters.

While no prior studies have generated disease clusters in SLD population, similar disease clusters have been reported in other populations. However, methodological differences, including variations in disease definitions, population characteristics and data sources, have led to some discrepancies in reported studies [25]. A systematic review [25] highlighted the substantial variability in multimorbidity clusters across studies, but noted three common clusters: mental health, cardiometabolic and respiratory, all of which emerged in this study.

Comparisons with previous studies reveal both similarities and differences. Krauth et al. [12] identified respiratory, mental health, cancer and cardiometabolic clusters, which were similar to our findings. Their cancer cluster was characterised with cancer, arthritis and thyroid diseases, corresponding to our cancer/osteoarthritis cluster and thyroid cluster in females. However, they did not examine sex differences. Calvin et al. [11] identified 7 clusters in females and 6 in males, including respiratory and cancer clusters in both sexes, and a thyroid cluster unique to females. Additionally, their cardiometabolic cluster, comprising hypertension, diabetes, coronary heart disease and stroke (in males only), closely resembled our cardiovascular clusters. Unlike their study, we excluded hypertension and diabetes due to their intrinsic association with SLD, but still observed high prevalence of these conditions in the cardiovascular clusters. Both Calvin et al. [11] and Krauth et al. [12] used only baseline self-report data in UK Biobank, potentially missing diagnoses captured in hospital records data.

Findings from broader UK-based studies further support our results, with mental health, cancer/osteoarthritis and stroke clusters repeatedly reported [26, 27]. Outside UK, disease clusters differ by population. Guisado-Clavero et al. [28] found 5 clusters in a Spanish population: musculoskeletal, endocrine-metabolic, digestive, neurological and cardiovascular clusters. A Chilean study [29] identified a broad depression/CVD/cancer cluster. A Chinese study [30] manually identified clusters including hypertension, diabetes, coronary heart disease, COPD and stroke.

The cancer/osteoarthritis cluster observed in both males and females aligns with findings from Krauth et al. [12], but whether this reflects shared risk factors (e.g. ageing) or common biological mechanisms remains uncertain. Prior research offers mixed evidence regarding this association. Ward and Alehashemi observed that osteoarthritis was associated with higher risk of breast, uterus and prostate cancers [31], but Beydon et al. indicated higher risk of lung, bladder, uterus and prostate cancers, but lower risks of cancers of pancreas and breast [32]. In contrast, Turkiewicz et al. found no association between osteoarthritis and most cancers, except for a negative association with colorectal cancer [33]. Mendelian randomisation studies suggested a positive association between osteoarthritis and bladder cancer [34], and ovarian and breast cancers in females [35]. Notably, most prior research was conducted in general populations, leaving the interaction between SLD, osteoarthritis and cancer risk largely unexplored. Further investigations are needed to clarify these associations and underlying mechanisms, which may be due to chronic inflammation, ageing and metabolic dysregulation. Given the limited research on this association in SLD populations, further investigation is warranted to clarify potential mechanisms.

Sex differences in cluster distribution were also evident, particularly in cardiovascular and mental health clusters. Males were significantly more likely to fall into cardiovascular clusters than females, with 28.4% of males assigned to these clusters (20.3% and 8.1% to the heart and stroke clusters, respectively), compared to 11.0% of females. This aligns with established evidence that males have higher CVD burden than females, driven by lifestyle and hormonal differences [36], and this sex disparity in CVD is projected to continue widening [37], further emphasising the importance of considering sex difference in CVD management [38, 39]. A mental health cluster characterised by depression and anxiety was identified in both males and females, but substance use disorder was prevalent only in males, consistent with evidence indicating a significantly higher burden of substance use disorder in males than in females [40]. A thyroid cluster specifically in females rather than in males reflects a higher prevalence of thyroid disorders in females [41].

The characteristics of disease clusters were further influenced by age, ethnicity, socioeconomic status and lifestyle factors. Individuals in the mental health and respiratory clusters were generally younger than those in cancer or cardiovascular clusters, likely reflecting the earlier onset of mental health and respiratory conditions compared to cancers and CVDs. Socioeconomic disparities were also evident, with the cancer and cardiovascular clusters being more prevalent among individuals with lower education attainment and greater socioeconomic deprivation. Additionally, we observed ethnic differences in cluster distribution, with White ethnicity more likely to fall into the mental health cluster [42–44].

MM was strongly linked to increased mortality, a finding consistent with prior studies [9, 45]. Research has also highlighted the contribution of MM to increased risk of dementia [11] and cancers [46]. Our study complemented the existing evidence by showing that extrahepatic MM increased mortality of CVD, extrahepatic cancers and liver-related diseases in people with SLD.

Cardiovascular-related clusters carried the highest mortality risk, underscoring the disproportionate burden of cardiovascular multimorbidity. This finding aligns with previous research. For example, a Hong Kong study [47] found that cardiovascular and cardiometabolic clusters had substantially higher mortality rate among eight clusters. Swain et al. [48] found that cardiovascular-musculoskeletal cluster was associated with the highest mortality, GP consultations and hospitalisation rate, followed by the cardiovascular cluster, among the five clusters. Krauth et al. [12] found increased mortality in the cardiometabolic and cancer clusters in UK Biobank. Collectively, these findings reinforce the critical tole of cardiovascular diseases in driving excess mortality, emphasising the need for early intervention and targeted management strategies to mitigate the risk.

The strong association between multimorbidity and mortality highlights the urgent need for a more integrated approach to SLD management. Instead of addressing conditions in isolation, care pathways must be adapted to account for the cumulative burden of multiple diseases. The identification of distinct disease clusters supports a more targeted approach to multimorbidity management. For instance, individuals in cardiovascular clusters—who face the highest mortality risk—may benefit from early and intensive cardiovascular risk management, including aggressive lipid and blood pressure control, lifestyle interventions and close monitoring for cardiac events.

Exploring sex differences in clustering patterns and outcomes is important. In our study, the clustering was conducted separately for males and females, and the latent classes are defined independently in each sex. Therefore, the resulting clusters are not directly comparable between sexes. Even if they share similar labels (e.g. respiratory cluster, mental health cluster), the underlying disease profiles and prevalences differ. Therefore, direct statistical testing of between-sex differences in cluster structure is neither straightforward nor necessarily meaningful. Moreover, some clusters are sex-specific, such as the thyroid cluster in females, while others diverge in structure, for example, females had one broad cardiovascular cluster, whereas males had two separate cluster for heart disease and stroke. This asymmetry precluded a valid interaction analysis between sex and cluster membership.

The disease clusters identified in this study offer important clinical implications for individuals with SLD. First, they provide a framework for risk stratification, as distinct clusters were associated with different mortality risks, with cardiovascular-related clusters showing the highest mortality. This can help clinicians identify high-risk subgroups, such as those with cardiovascular risk factors or diseases, and tailor management accordingly to reduce cardiovascular comorbidities. Second, the clusters support personalised care pathways, by highlighting common co-occurring conditions that may benefit from integrated services (e.g. joint cardiology and liver, or joint psychiatry and liver clinics). Third, these clusters align with and extend known multimorbidity patterns in chronic diseases, including previously described associations between SLD and cardiometabolic conditions [4, 5]; therefore, cardiometabolic comorbidity prevention and liver progression should be emphasised. The presence of sex-specific clusters (e.g. a thyroid cluster in females and differentiated CVD clusters in males) underscores the need for sex-sensitive approaches in prevention and care. Additionally, understanding the composition and outcomes of these clusters may inform healthcare planning and resource allocation, particularly in settings with a high burden of multimorbidity. Overall, these findings support the potential of multimorbidity clustering to enhance clinical decision-making and improve outcomes in people with SLD.

This study has several strengths, including a large sample size, long follow-up period and comprehensive multimorbidity phenotyping. We also conducted various sensitivity analyses and confirmed the stability of the clustering process and validity of the derived clusters and cluster assignments. However, several limitations should be acknowledged. First, we used the FLI to indicate liver steatosis rather than imaging or histological evidence, which may introduce misclassification bias; however, FLI has been widely used as a non-invasive indicator for liver steatosis. Second, our multimorbidity phenotyping relied on self-reported data and hospital records, excluding primary care data, potentially leading to an underrepresentation of milder disease cases. This may introduce misclassification bias. We defined comorbidity based on validated codelists and used algorithms consistent with previous published work using UK Biobank [49, 50]. Third, UK Biobank participants tend to be predominantly White, less socioeconomically deprived and healthier than the general population [51], limiting the generalisability of our findings to more diverse or disadvantaged populations. Lastly, we did not account for the temporal sequence of disease development, which may influence the clustering patterns observed.

## Conclusions

In this large cohort study of individuals with SLD and extrahepatic multimorbidity, we identified distinct disease clusters that differed by sex and were associated with varying mortality risks. Cardiovascular-related clusters had the highest mortality, highlighting the need for targeted prevention and management strategies. Our findings underscore the complex interplay between multimorbidity and SLD, emphasising the importance of a holistic, patient-centred approach to risk stratification and clinical care. Further research is needed to explore the underlying mechanisms and potential interventions to mitigate the burden of multimorbidity in this population.

## Supplementary Information

Additional file 1: Supplementary methods. Figures S1–S3. Tables S1–S10. Fig. S1. Sample size-adjusted Bayesian information criterion (aBIC) versus number of clusters in latent class analyses. Fig. S2. Distributions of derived clusters in people with steatotic liver disease. Fig. S3. Causes of death in males and females with steatotic liver disease and multimorbidity, stratified by disease clusters. Table S1. Number of clusters and model performance measures in latent class analyses. Table S2. List of conditions in each cluster that had a probability > 5% and higher within-cluster prevalence than overall prevalence. Table S3. Baseline characteristics of males with steatotic liver disease, stratified by disease clusters. Table S4. Baseline characteristics of females with steatotic liver disease, stratified by disease clusters. Table S5. Associations between disease clusters and all-cause mortality in males and females with steatotic liver disease. Table S6. Sensitivity analysis for associations between disease clusters and all-cause mortality in males and females with steatotic liver disease. Table S7. Latent class analysis derived disease clusters in males and females with steatotic liver disease in sensitivity analyses of random samples of 80% and 50% of total sample. Table S8. Posterior probability in each cluster derived from full, 80% and 50% samples using latent class analysis (showing median (interquartile interval)). Table S9. Proportion of people with posterior probability < 70%. Table S10. Associations (HR (95%CI)) between the disease clusters and all-cause mortality, and mortality of cardiovascular diseases and extrahepatic cancers, hepatocellular carcinoma and liver-related diseases in males and females with SLD in sensitivity analysis removing individuals with posterior probability < 70%.

## Data Availability

UK Biobank data are available to registered researchers at https://www.ukbiobank.ac.uk/.

## Abbreviations

SLD: Steatotic liver disease
CVD: Cardiovascular disease
HR: Hazard ratio
CI: Confidence interval
HCC: Hepatocellular carcinoma
MM: Multimorbidity
LTC: Long-term condition
FLI: Fatty liver index
BMI: Body mass index
TG: Triglycerides
GGT: Gamma-glutamyl transferase
MASLD: Dysfunction-associated steatotic liver disease
MetALD: Metabolic dysfunction and alcohol related liver disease
ALD: Alcohol related liver disease
CMRF: Cardiometabolic risk factors
HDL: High-density lipoprotein
LCA: Latent class analysis
BIC: Bayesian information criterion
aBIC: Adjusted Bayesian information criterion
AIC: Akaike information criterion
O/E ratio: Observed/expected prevalence ratio
