# Supplementary material for: Extrahepatic disease clusters and mortality in people with steatotic liver diseases: a prospective analysis of 64,749 females and 113,587 males in the UK Biobank
Source: BMC Med. 2025 Jul 31;23:450. doi: 10.1186/s12916-025-04288-4 (PMC12315339; doi:10.1186/s12916-025-04288-4)
Supplement: Supplementary file 1 — Additional file 1: Supplementary methods. Figures S1–S3. Tables S1–S10. Fig. S1. Sample size-adjusted Bayesian information criterion (aBIC) versus number of clusters in latent class analyses. Fig. S2. Distributions of derived clusters in people with steatotic liver disease. Fig. S3. Causes of death in males and females with steatotic liver disease and multimorbidity, stratified by disease clusters. Table S1. Number of clusters and model performance measures in latent class analyses. Table S2. List of conditions in each cluster that had a probability > 5% and higher within-cluster prevalence than overall prevalence. Table S3. Baseline characteristics of males with steatotic liver disease, stratified by disease clusters. Table S4. Baseline characteristics of females with steatotic liver disease, stratified by disease clusters. Table S5. Associations between disease clusters and all-cause mortality in males and females with steatotic liver disease. Table S6. Sensitivity analysis for associations between disease clusters and all-cause mortality in males and females with steatotic liver disease. Table S7. Latent class analysis derived disease clusters in males and females with steatotic liver disease in sensitivity analyses of random samples of 80% and 50% of total sample. Table S8. Posterior probability in each cluster derived from full, 80% and 50% samples using latent class analysis (showing median (interquartile interval)). Table S9. Proportion of people with posterior probability < 70%. Table S10. Associations (HR (95%CI)) between the disease clusters and all-cause mortality, and mortality of cardiovascular diseases and extrahepatic cancers, hepatocellular carcinoma and liver-related diseases in males and females with SLD in sensitivity analysis removing individuals with posterior probability < 70%. [file 12916_2025_4288_MOESM1_ESM.docx]

Extrahepatic disease clusters and mortality in people with steatotic liver diseases: a prospective analysis of 64749 females and 113587 males in the UK Biobank

Supplementary materials

Contents

[Supplementary methods 2](#_Toc203658145)

[*Figure S1: Sample size-adjusted Bayesian Information Criterion (aBIC) versus number of clusters in latent class analyses.* 3](#_Toc203658146)

[*Table S1: Number of clusters and model performance measures in latent class analyses* 4](#_Toc203658147)

[*Table S2: list of conditions in each cluster that had a probability>5% and higher within-cluster prevalence than overall prevalence* 5](#_Toc203658148)

[*Figure S2: Distributions of derived clusters in people with steatotic liver disease* 7](#_Toc203658149)

[*Table S3: Baseline characteristics of males with steatotic liver disease, stratified by disease clusters* 8](#_Toc203658150)

[*Table S4: Baseline characteristics of females with steatotic liver disease, stratified by disease clusters* 9](#_Toc203658151)

[*Table S5: Associations between disease clusters and all-cause mortality in males and females with steatotic liver disease* 11](#_Toc203658152)

[*Table S6: Sensitivity analysis for associations between disease clusters and all-cause mortality in males and females with steatotic liver disease* 12](#_Toc203658153)

[*Figure S3: Causes of death in males and females with steatotic liver disease and multimorbidity, stratified by disease clusters* 13](#_Toc203658154)

[Table S7: Latent class analysis derived disease clusters in males and females with steatotic liver disease in sensitivity analyses of random samples of 80% and 50% of total sample 14](#_Toc203658155)

[Table S8: Posterior probability in each cluster derived from full, 80% and 50% samples using latent class analysis (showing median (interquartile interval) 15](#_Toc203658156)

[*Table S9: proportion of people with posterior probability < 70%* 16](#_Toc203658157)

[*Table S10: Associations (HR (95%CI)) between the disease clusters and all-cause mortality, and mortality of cardiovascular diseases and extrahepatic cancers, hepatocellular carcinoma and liver related diseases in males and females with SLD in sensitivity analysis removing individuals with posterior probability < 70%* 17](#_Toc203658158)

## Supplementary methods

The codelists for chronic liver diseases and long-term conditions for defining multimorbidity can be found online: [codelists.xlsx](https://docs.google.com/spreadsheets/d/1pErQT3krBj3ZpVk2JhM5styq_MYxzQOl/edit?usp=sharing&ouid=108654502587298592146&rtpof=true&sd=true)

| (A) primary analysis in full sample |
| --- |
| 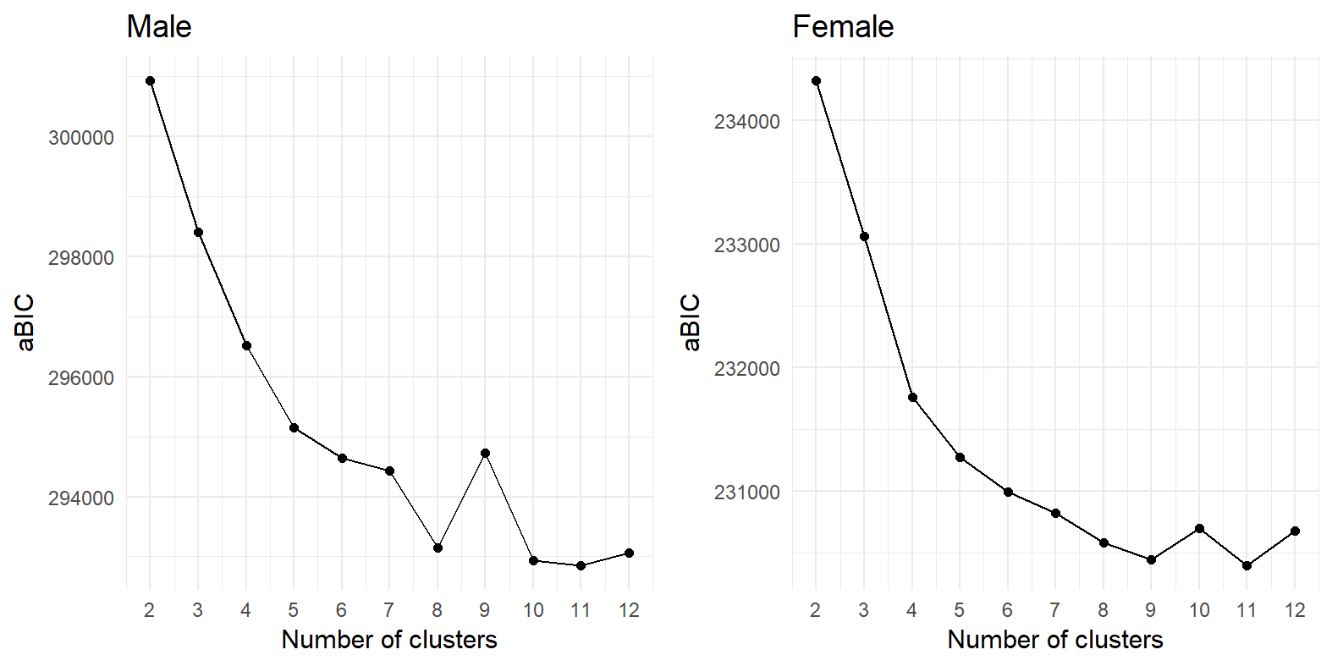 |
| (B) sensitivity analysis in a random 80% sample |
| 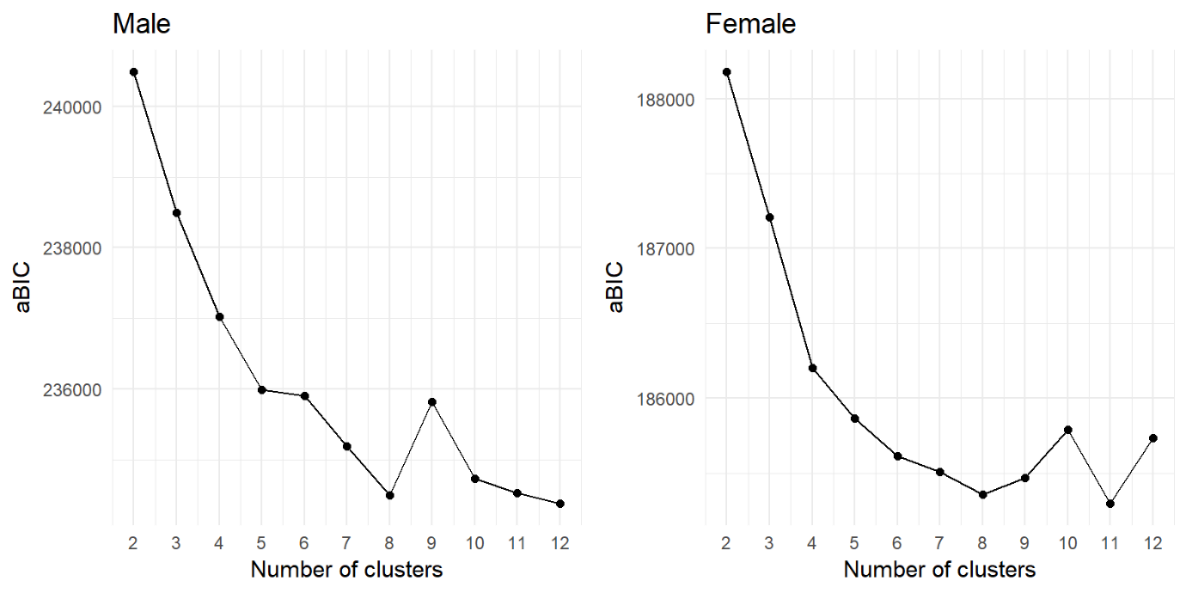 |
| (C) sensitivity analysis in a random 50% sample |
| 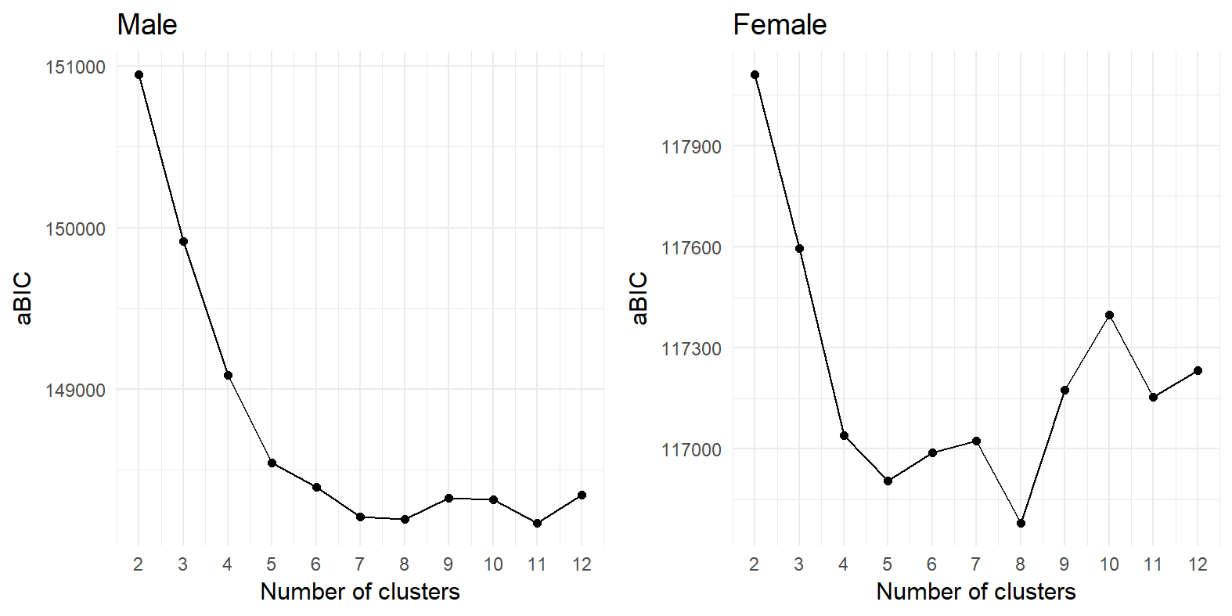 |

### Figure S1: Sample size-adjusted Bayesian Information Criterion (aBIC) versus number of clusters in latent class analyses.

### Table S1: Number of clusters and model performance measures in latent class analyses

| Number of clusters | AIC | BIC | aBIC | Proportion (%) of the smallest cluster |
| --- | --- | --- | --- | --- |
| Males |  |  |  |  |
| 2 | 300491.748 | 301227.619 | 300932.07 | 39.4 |
| 3 | 297741.324 | 298849.088 | 298404.174 | 27.6 |
| 4 | 295628.949 | 297108.604 | 296514.327 | 14.6 |
| 5 | 294033.792 | 295885.339 | 295141.698 | 8.1 |
| 6 | 293313.228 | 295536.667 | 294643.662 | 8.0 |
| 7 | 292870.063 | 295465.395 | 294423.025 | 1.3 |
| 8 | 291376.088 | 294343.312 | 293151.578 | 7.3 |
| 9 | 292725.985 | 296065.100 | 294724.003 | <0.1 |
| 10 | 290714.997 | 294426.004 | 292935.544 | 2.8 |
| 11 | 290406.877 | 294489.777 | 292849.952 | 1.8 |
| 12 | 290394.775 | 294849.566 | 293060.378 | 2.1 |
| Females |  |  |  |  |
| 2 | 233895.412 | 234623.964 | 234322.061 | 19.0 |
| 3 | 232419.509 | 233516.171 | 233061.727 | 15.9 |
| 4 | 230897.586 | 232362.358 | 231755.374 | 11.2 |
| 5 | 230195.111 | 232027.994 | 231268.469 | 11.0 |
| 6 | 229698.117 | 231899.110 | 230987.045 | 4.0 |
| 7 | 229313.795 | 231882.899 | 230818.293 | 4.2 |
| 8 | 228859.499 | 231796.713 | 230579.567 | 1.7 |
| 9 | 228505.051 | 231810.376 | 230440.689 | 3.9 |
| 10 | 228541.020 | 232214.455 | 230692.228 | 1.2 |
| 11 | 228026.244 | 232067.79 | 230393.022 | 0.7 |
| 12 | 228093.387 | 232503.043 | 230675.735 | 2.4 |

AIC: Akaike information criterion. BIC: Bayern information criterion. aBIC: sample size adjusted BIC.

### Table S2: list of conditions in each cluster that had a probability>5% and higher within-cluster prevalence than overall prevalence

| Cluster | Condition | Probability,  % | Prevalence within the cluster, /100 | Prevalence in the sample,/100 | E/O ratio | Exclusivity, % |
| --- | --- | --- | --- | --- | --- | --- |
| Males |  |  |  |  |  |  |
| Respiratory | Asthma | 100 | 100 | 32.4 | 3.1 | 85.3 |
|  | Chronic respiratory disease | 46.7 | 43 | 30.1 | 1.4 | 39.5 |
|  | COPD | 16.5 | 15.6 | 10.6 | 1.5 | 40.9 |
| Cancer  /osteoarthritis | Chronic respiratory disease | 36.9 | 40.6 | 30.1 | 1.3 | 40.4 |
|  | Solid organ cancers | 27.3 | 29.4 | 12.4 | 2.4 | 71.0 |
|  | Osteoarthritis | 22 | 23.9 | 12 | 2.0 | 60.0 |
|  | Thyroid disorder | 10.1 | 9.5 | 7 | 1.4 | 40.6 |
|  | Connective tissue disease | 8.3 | 8.9 | 4.1 | 2.1 | 64.2 |
|  | Venous thromboembolism | 7.7 | 7.6 | 4.4 | 1.7 | 52.3 |
|  | Osteoporosis | 6.1 | 6 | 2.7 | 2.2 | 67.2 |
|  | Inflammatory bowel disease | 6 | 6.6 | 3.3 | 2.0 | 59.8 |
| Stroke | Stroke | 91.6 | 98.2 | 9.4 | 10.4 | 84.1 |
|  | Ischemic heart disease | 34.9 | 34.3 | 33.8 | 1.0 | 8.2 |
|  | Paralysis | 16.7 | 15.1 | 1.5 | 9.8 | 79.1 |
|  | TIA | 13.9 | 12.1 | 1.6 | 7.5 | 60.4 |
|  | Epilepsy | 11.1 | 10.2 | 4.1 | 2.5 | 20.0 |
|  | Substance use disorder | 9.1 | 8.6 | 6.7 | 1.3 | 10.4 |
|  | Venous thromboembolism | 5.2 | 5 | 4.4 | 1.1 | 9.3 |
| Mental health | Depression | 69.7 | 79.8 | 17.3 | 4.6 | 64.4 |
|  | Anxiety | 24.1 | 27.8 | 5.3 | 5.3 | 73.5 |
|  | Substance use disorder | 18.4 | 18.7 | 6.7 | 2.8 | 38.8 |
|  | Schizophrenia | 8.1 | 9.3 | 1.3 | 6.9 | 96.3 |
|  | Bipolar disorder | 7.4 | 8.6 | 1.4 | 5.9 | 82.9 |
|  | Epilepsy | 6.7 | 6.8 | 4.1 | 1.6 | 22.8 |
| Heart | Ischemic heart disease | 77.1 | 83.3 | 33.8 | 2.5 | 50.0 |
|  | Arrythmia | 44.4 | 52.7 | 16.2 | 3.3 | 65.9 |
|  | Heart failure | 21.4 | 25.5 | 5.3 | 4.8 | 96.5 |
|  | Heart valve disorder | 15.7 | 18.9 | 5.0 | 3.8 | 77.2 |
|  | COPD | 10.3 | 10.8 | 10.6 | 1.0 | 20.7 |
|  | Thyroid disorder | 7.5 | 9 | 7 | 1.3 | 26.0 |
|  | Substance use disorder | 7 | 9 | 6.7 | 1.3 | 27.1 |
|  | Chronic kidney disease | 5.5 | 6.5 | 3.3 | 2.0 | 39.7 |
| Females |  |  |  |  |  |  |
| Respiratory | Asthma | 100 | 100 | 39.2 | 2.6 | 66.4 |
|  | Chronic respiratory disease | 36.6 | 40.5 | 25.7 | 1.6 | 40.9 |
|  | COPD | 14.9 | 17.2 | 8.6 | 2.0 | 52.3 |
| Thyroid disorder | Thyroid disorder | 100 | 100 | 28 | 3.6 | 69.9 |
|  | Connective tissue disease | 7.9 | 8 | 7.9 | 1.0 | 19.6 |
| Cancer  /osteoarthritis | Chronic respiratory disease | 33.6 | 34.5 | 25.7 | 1.3 | 28.8 |
|  | Solid organ cancers | 30.9 | 30.9 | 14.7 | 2.1 | 45.1 |
|  | Osteoarthritis | 21.9 | 24 | 13.4 | 1.8 | 38.5 |
|  | Endometriosis | 13.8 | 13.8 | 7.3 | 1.9 | 40.4 |
|  | Connective tissue disease | 12.3 | 12.2 | 7.9 | 1.5 | 33.0 |
|  | Osteoporosis | 12.1 | 12.5 | 6.4 | 2.0 | 42.3 |
|  | Venous thromboembolism | 7.4 | 7.2 | 4.6 | 1.6 | 33.6 |
|  | Inflammatory bowel disease | 5.6 | 5.8 | 2.5 | 2.3 | 48.8 |
|  | Epilepsy | 5.2 | 5.3 | 3.4 | 1.5 | 33.0 |
| Mental health | Depression | 100 | 100 | 26.1 | 3.8 | 84.1 |
|  | Anxiety | 16.3 | 14.4 | 6.7 | 2.2 | 47.3 |
| Heart/stroke | Ischemic heart disease | 51.7 | 63.4 | 16.3 | 3.9 | 42.8 |
|  | Arrythmia | 26.4 | 33.7 | 7.5 | 4.5 | 49.2 |
|  | Stroke | 24.7 | 32.6 | 6 | 5.4 | 59.8 |
|  | Heart valve disorder | 14.4 | 18.9 | 3.5 | 5.5 | 60.2 |
|  | COPD | 12.1 | 13 | 8.6 | 1.5 | 16.7 |
|  | Heart failure | 10 | 14 | 1.6 | 8.7 | 96.1 |
|  | Connective tissue disease | 8.3 | 8.5 | 7.9 | 1.1 | 11.9 |
|  | Venous thromboembolism | 7.1 | 8.3 | 4.6 | 1.8 | 19.9 |
|  | Paralysis | 6.2 | 8.8 | 1.2 | 7.4 | 81.8 |
|  | Vision impairment | 6.2 | 7.5 | 2.5 | 3.1 | 33.8 |

E/O ratio: disease prevalence in the cluster divided by the disease prevalence in the overall sample for latent class analysis. Exclusivity: the number of participants with the disease in the cluster divided by the number of participants with the disease in the overall sample for latent class analysis.

|  |  |
| --- | --- |

### Figure S2: Distributions of derived clusters in people with steatotic liver disease

MASLD: dysfunction-associated steatotic liver disease. MetALD: metabolic dysfunction and alcohol related liver disease. ALD: alcohol related liver disease. The disease clusters were derived via latent class analysis.

### Table S3: Baseline characteristics of males with steatotic liver disease, stratified by disease clusters

|  | Respiratory cluster | Mental health cluster | Cancer/ osteoarthritis cluster | Stroke cluster | Heart cluster | Overall |
| --- | --- | --- | --- | --- | --- | --- |
|  | n = 5587 | n = 2814 | n = 6060 | n = 1632 | n = 4090 | n = 20183 |
| Age, years | 57.4 (8.1) | 56.0 (7.7) | 60.7 (6.9) | 60.9 (6.7) | 62.0 (6.0) | 59.4 (7.5) |
| Townsend deprivation index |  |  |  |  |  |  |
| 1st fifth (least deprived) | 1040 (18.6%) | 327 (11.6%) | 975 (16.1%) | 209 (12.8%) | 637 (15.6%) | 3188 (15.8%) |
| 5th fifth (most deprived) | 1387 (24.8%) | 1062 (37.7%) | 1499 (24.7%) | 577 (35.4%) | 1203 (29.4%) | 5728 (28.4%) |
| Education, higher education | 1577 (28.2%) | 732 (26.0%) | 1346 (22.2%) | 293 (18.0%) | 779 (19.0%) | 4727 (23.4%) |
| Ethnicity, White | 5332 (95.4%) | 2682 (95.3%) | 5786 (95.5%) | 1549 (94.9%) | 3914 (95.7%) | 19263 (95.4%) |
| Smoking, never | 2374 (42.5%) | 986 (35.0%) | 2200 (36.3%) | 512 (31.4%) | 1182 (28.9%) | 7254 (35.9%) |
| Physical activity, high | 1470 (26.3%) | 674 (24.0%) | 1634 (27.0%) | 339 (20.8%) | 1025 (25.1%) | 5142 (25.5%) |
| BMI, kg/m^2^ | 30.9 (4.3) | 31.0 (4.7) | 30.9 (4.2) | 31.2 (4.4) | 31.5 (4.5) | 31.1 (4.4) |
| Waist circumference, cm | 106.0 (10.6) | 106.0 (11.1) | 105.8 (10.4) | 106.8 (10.8) | 107.6 (11.2) | 106.4 (10.8) |
| Systolic blood pressure, mmHg | 142.6 (16.2) | 139.3 (16.5) | 143.0 (17.4) | 141.6 (18.5) | 138.4 (19.4) | 141.3 (17.6) |
| Diastolic blood pressure, mmHg | 85.6 (9.6) | 84.8 (10.1) | 84.2 (10.1) | 83.4 (10.3) | 80.5 (11.0) | 83.9 (10.3) |
| ALT, log_10_ U/L | 1.5 (0.2) | 1.5 (0.2) | 1.4 (0.2) | 1.4 (0.2) | 1.4 (0.2) | 1.4 (0.2) |
| AST, log_10_ U/L | 1.4 (0.2) | 1.5 (0.2) | 1.4 (0.2) | 1.4 (0.2) | 1.5 (0.2) | 1.4 (0.2) |
| GGT, log_10_ U/L | 1.7 (0.3) | 1.7 (0.3) | 1.7 (0.3) | 1.7 (0.3) | 1.7 (0.3) | 1.7 (0.3) |
| Triglycerides, mmol/L | 2.2 [1.4] | 2.3 [1.6] | 2.1 [1.4] | 2.1 [1.3] | 2.0 [1.4] | 2.1 [1.4] |
| HDL cholesterol, mmol/L | 1.1 (0.4) | 1.1 (0.4) | 1.1 (0.4) | 1.1 (0.4) | 1.0 (0.4) | 1.1 (0.4) |
| HbA1c, mmol/mol | 37.4 (10.9) | 37.7 (10.8) | 37.7 (10.8) | 39.8 (11.8) | 41.1 (13.4) | 38.5 (11.6) |
| Platelet, 10^9^ cells/L | 245.9 (59.9) | 242.6 (59.1) | 235.0 (61.0) | 238.8 (64.5) | 226.6 (60.0) | 237.7 (60.9) |
| Hypertension | 4539 (81.2%) | 2103 (74.7%) | 4809 (79.4%) | 1220 (74.8%) | 2779 (67.9%) | 15450 (76.5%) |
| Obesity | 5490 (98.3%) | 2742 (97.4%) | 5942 (98.1%) | 1591 (97.5%) | 4023 (98.4%) | 19788 (98.0%) |
| Diabetes | 1780 (31.9%) | 995 (35.4%) | 2157 (35.6%) | 776 (47.5%) | 2087 (51.0%) | 7795 (38.6%) |
| High triglycerides | 3872 (69.3%) | 2061 (73.2%) | 4160 (68.6%) | 1056 (64.7%) | 2610 (63.8%) | 13759 (68.2%) |
| Low HDL-cholesterol | 1691 (30.3%) | 1029 (36.6%) | 2115 (34.9%) | 641 (39.3%) | 1790 (43.8%) | 7266 (36.0%) |

BMI: body mass index. FIB4: fibrosis-4 score. ALT: alanine aminotransferase. AST: aspartate aminotransferase. GGT: gamma-glutamyl transferase. HDL: high-density lipoprotein. HbA1c: glycated hemoglobin.

### Table S4: Baseline characteristics of females with steatotic liver disease, stratified by disease clusters

|  | Respiratory cluster | Mental health cluster | Cancer/osteoarthritis cluster | Heart/stroke cluster | Thyroid cluster | Overall |
| --- | --- | --- | --- | --- | --- | --- |
|  | n = 4116 | n = 3469 | n = 3396 | n = 1745 | n = 3093 | n = 15819 |
| Age, years | 57.8 (7.6) | 55.8 (7.6) | 59.9 (6.8) | 61.2 (6.6) | 59.8 (6.7) | 58.6 (7.4) |
| Townsend deprivation index |  |  |  |  |  |  |
| 1st fifth (least deprived) | 548 (13.3%) | 456 (13.1%) | 534 (15.7%) | 184 (10.5%) | 466 (15.1%) | 2188 (13.8%) |
| 5th fifth (most deprived) | 1304 (31.7%) | 1169 (33.7%) | 916 (27.0%) | 650 (37.2%) | 814 (26.3%) | 4853 (30.7%) |
| Education, Higher education | 907 (22.0%) | 776 (22.4%) | 648 (19.1%) | 251 (14.4%) | 575 (18.6%) | 3157 (20.0%) |
| Ethnicity, White | 3793 (92.2%) | 3298 (95.1%) | 3186 (93.8%) | 1643 (94.2%) | 2933 (94.8%) | 14853 (93.9%) |
| Smoking, never | 2147 (52.2%) | 1735 (50.0%) | 1760 (51.8%) | 742 (42.5%) | 1599 (51.7%) | 7983 (50.5%) |
| Physical activity, high | 819 (19.9%) | 632 (18.2%) | 678 (20.0%) | 297 (17.0%) | 616 (19.9%) | 3042 (19.2%) |
| BMI, kg/m2 | 34.3 (5.4) | 34.3 (5.7) | 33.0 (4.8) | 33.8 (5.4) | 33.9 (5.1) | 33.9 (5.3) |
| Waist circumference, cm | 102.6 (10.9) | 102.4 (11.4) | 99.9 (10.1) | 102.3 (11.2) | 101.4 (10.4) | 101.7 (10.8) |
| Systolic blood pressure, mmHg | 140.4 (17.7) | 136.1 (17.0) | 141.2 (18.4) | 138.8 (18.9) | 141.0 (17.9) | 139.6 (18.0) |
| Diastolic blood pressure, mmHg | 84.6 (9.7) | 83.8 (9.6) | 83.7 (9.8) | 80.5 (11.0) | 83.9 (9.7) | 83.6 (9.9) |
| ALT, log_10_ U/L | 1.4 (0.2) | 1.4 (0.2) | 1.4 (0.2) | 1.3 (0.2) | 1.4 (0.2) | 1.4 (0.2) |
| AST, log_10_ U/L | 1.4 (0.2) | 1.4 (0.2) | 1.4 (0.2) | 1.4 (0.2) | 1.4 (0.2) | 1.4 (0.2) |
| GGT, log_10_ U/L | 1.6 (0.3) | 1.6 (0.3) | 1.6 (0.3) | 1.6 (0.3) | 1.6 (0.3) | 1.6 (0.3) |
| Triglycerides, mmol/L | 2.0 [1.2] | 2.1 [1.3] | 2.1 [1.2] | 2.0 [1.3] | 2.0 [1.2] | 2.0 [1.2] |
| HDL cholesterol, mmol/L | 1.3 (0.5) | 1.2 (0.4) | 1.3 (0.4) | 1.2 (0.5) | 1.2 (0.4) | 1.3 (0.4) |
| HbA1c, mmol/mol | 37.7 (10.2) | 37.3 (9.8) | 37.7 (10.5) | 41.2 (12.9) | 38.4 (10.5) | 38.1 (10.6) |
| Platelet, 10^9^ cells/L | 283.2 (66.9) | 281.6 (65.1) | 272.4 (66.5) | 270.8 (70.5) | 272.8 (63.1) | 277.1 (66.3) |
| Hypertension | 3408 (82.8%) | 2649 (76.4%) | 2836 (83.5%) | 1551 (88.9%) | 2594 (83.9%) | 13038 (82.4%) |
| Obesity | 4112 (99.9%) | 3460 (99.7%) | 3391 (99.9%) | 1737 (99.5%) | 3088 (99.8%) | 15788 (99.8%) |
| Diabetes | 1567 (38.1%) | 1190 (34.3%) | 1257 (37.0%) | 936 (53.6%) | 1232 (39.8%) | 6182 (39.1%) |
| High triglycerides | 2979 (72.4%) | 2671 (77.0%) | 2645 (77.9%) | 1575 (90.3%) | 2417 (78.1%) | 12287 (77.7%) |
| Low HDL-cholesterol | 2550 (62.0%) | 2265 (65.3%) | 2149 (63.3%) | 1505 (86.2%) | 2037 (65.9%) | 10506 (66.4%) |

BMI: body mass index. FIB4: fibrosis-4 score. ALT: alanine aminotransferase. AST: aspartate aminotransferase. GGT: gamma-glutamyl transferase. HDL: high-density lipoprotein. HbA1c: glycated hemoglobin.

### Table S5: Associations between disease clusters and all-cause mortality in males and females with steatotic liver disease

| Group | Events/total | Mortality rate  (per 1000 person-year) | HR (95%CI) |
| --- | --- | --- | --- |
| **Males** |  |  |  |
| No MM | 9601 / 93404 | 7.7 | Reference |
| MM | 4994 / 20183 | 20.0 | 2.00 (1.93, 2.08) |
| Respiratory cluster | 975 / 5587 | 13.5 | 1.62 (1.51, 1.73) |
| Mental health cluster | 578 / 2814 | 16.2 | 1.84 (1.69, 2.00) |
| Cancer/osteoarthritis cluster | 1463 / 6060 | 19.4 | 1.85 (1.75, 1.96) |
| Stroke cluster | 529 / 1632 | 26.9 | 2.36 (2.16, 2.58) |
| Heart cluster | 1449 / 4090 | 30.1 | 2.63 (2.48, 2.78) |
| **Females** |  |  |  |
| No MM | 3747 / 48930 | 5.7 | Reference |
| MM | 2424 / 15819 | 11.7 | 1.80 (1.71, 1.90) |
| Respiratory cluster | 573 / 4116 | 10.6 | 1.73 (1.58, 1.89) |
| Mental health cluster | 419 / 3469 | 9.1 | 1.57 (1.42, 1.74) |
| Cancer/osteoarthritis cluster | 549 / 3396 | 12.5 | 1.85 (1.69, 2.02) |
| Heart/stroke cluster | 492 / 1745 | 22.7 | 2.90 (2.64, 3.20) |
| Thyroid cluster | 391 / 3093 | 9.6 | 1.42 (1.28, 1.58) |

Model was stratified by region and age groups, adjusted for ethnic, education, deprivation, physical activity, alcohol intake, smoking. MM: multimorbidity. HR (95%CI): hazard ratio (95% confidence interval).

### Table S6: Sensitivity analysis for associations between disease clusters and all-cause mortality in males and females with steatotic liver disease

|  |  | HR (95%CI) | HR (95%CI) |
| --- | --- | --- | --- |
| Cluster | Events/total | Additional adjustment of CMRFs | Removing two years of follow up |
| Males |  |  |  |
| No MM | 9601 / 93404 | Reference | Reference |
| MM | 4994 / 2424 | 1.90 (1.84, 1.97) | 1.85 (1.79, 1.92) |
| Respiratory cluster | 975 / 5587 | 1.57 (1.47, 1.67) | 1.60 (1.50, 1.72) |
| Mental health cluster | 578 / 2814 | 1.77 (1.63, 1.93) | 1.77 (1.62, 1.93) |
| Cancer/osteoarthritis cluster | 1463 / 6060 | 1.80 (1.70, 1.90) | 1.80 (1.69, 1.90) |
| Stroke cluster | 529 / 1632 | 2.18 (2.00, 2.39) | 2.27 (2.07, 2.49) |
| Heart cluster | 1449 / 4090 | 2.41 (2.27, 2.55) | 2.54 (2.39, 2.69) |
| Females |  |  |  |
| No MM | 3747 / 48930 | Reference | Reference |
| MM | 2424 / 15819 | 1.74 (1.65, 1.84) | 1.70 (1.61, 1.80) |
| Respiratory cluster | 573 / 4116 | 1.68 (1.54, 1.84) | 1.71 (1.56, 1.87) |
| Mental health cluster | 419 / 3469 | 1.54 (1.39, 1.71) | 1.54 (1.39, 1.71) |
| Cancer/osteoarthritis cluster | 549 / 3396 | 1.82 (1.66, 1.99) | 1.78 (1.62, 1.95) |
| Heart/stroke cluster | 492 / 1745 | 2.64 (2.40, 2.91) | 2.84 (2.58, 3.14) |
| Thyroid cluster | 391 / 3093 | 1.37 (1.24, 1.53) | 1.39 (1.25, 1.55) |

MM: multimorbidity. CMRF: cardiometabolic risk factors (obesity, diabetes, hypertension, high TG and low HDL-cholesterol). HR (95%CI): hazard ratio (95% confidence interval).

### Figure S3: Causes of death in males and females with steatotic liver disease and multimorbidity, stratified by disease clusters

|  |  |
| --- | --- |

CVD: cardiovascular diseases.

### Table S7: Latent class analysis derived disease clusters in males and females with steatotic liver disease in sensitivity analyses of random samples of 80% and 50% of total sample

|  | Males |  | Females |
| --- | --- | --- | --- |
| **80% sample** | (n=16146) |  | (n=12655) |
| Cluster name | Respiratory cluster (n = 4475, 27.7%) |  | Respiratory cluster (n = 3239, 25.6%) |
| Characteristic diseases* | - Asthma (100%), - COPD (15%), - Other chronic respiratory diseases (43%) |  | - Asthma (100%), - COPD (17%), - Other chronic respiratory diseases (41%) |
| Cluster name | Mental health cluster (n = 2249, 13.9%) |  | Mental health cluster (n = 2824, 22.3%) |
| Characteristic diseases | - Depression (78%), - Anxiety (28%), - Substance use disorder (19%) |  | - Depression (100%), - Anxiety (14%) |
| Cluster name | Cancer/ osteoarthritis cluster (n = 4868, 30.1%) |  | Cancer/ osteoarthritis cluster (n = 2453, 19.4%) |
| Characteristic diseases | - Solid organ cancers (29%), - Other chronic respiratory diseases (41%), - Osteoarthritis (23%) |  | - Solid organ cancers (32%), - Other chronic respiratory disease (35%), - Osteoarthritis (21%) |
| Cluster name | Stroke cluster (n = 1277, 7.9%) |  | Heart/stroke cluster (n = 1530, 12.1%) |
| Characteristic diseases | - Stroke (98%), - Paralysis (15%) |  | - Ischemic heart disease (64%), - Arrythmia (31%), - Stroke (32%) |
| Cluster name | Heart cluster (n = 3277, 20.3%) |  | Thyroid cluster (n = 2609, 20.6%) |
| Characteristic diseases | - Ischemic heart disease (83%), - Arrythmia (53%), - Heart failure (26%) |  | - Thyroid disorder (100%) - Connective tissue diseases (8%) |
|  |  |  |  |
| **50% sample** | (n=10092) |  | (n=7910) |
| Cluster name | Respiratory cluster (n = 2604, 25.8%) |  | Respiratory cluster (n = 1955, 24.7%) |
| Characteristic diseases* | - Asthma (100%), - COPD (18%), - Other chronic respiratory diseases (46%) |  | - Asthma (100%), - COPD (16%), - Other chronic respiratory diseases (39%) |
| Cluster name | Mental health cluster (n = 1602, 15.9%) |  | Mental health cluster (n = 1683, 21.3%) |
| Characteristic diseases | - Depression (78%), - Anxiety (24%), - Substance use disorder (19%) |  | - Depression (99%), - Anxiety (16%) |
| Cluster name | Cancer/ osteoarthritis cluster (n = 3001, 29.7%) |  | Cancer/ osteoarthritis cluster (n = 1760, 22.2%) |
| Characteristic diseases | - Solid organ cancers (29%), - Other chronic respiratory diseases (40%), - Osteoarthritis (25%) |  | - Solid organ cancers (31%), - Other chronic respiratory disease (34%), - Osteoarthritis (24%) |
| Cluster name | Stroke cluster (n = 833, 8.3%) |  | Heart/stroke cluster (n = 837, 10.6%) |
| Characteristic diseases | - Stroke (97%), - Paralysis (16%) |  | - Ischemic heart disease (66%), - Arrythmia (34%), - Stroke (33%) |
| Cluster name | Heart cluster (n = 2052, 20.3%) |  | Thyroid cluster (n = 1675, 21.2%) |
| Characteristic diseases | - Ischemic heart disease (82%), - Arrythmia (54%), - Heart failure (25%) |  | - Thyroid disorder (100%) - Connective tissue diseases (8%) |

Showing the characteristic diseases and their prevalences in that cluster. COPD: chronic obstructive pulmonary disease.

### Table S8: Posterior probability in each cluster derived from full, 80% and 50% samples using latent class analysis (showing median (interquartile interval)

|  | Full sample | 80% sample | 50% sample |
| --- | --- | --- | --- |
| **Males** |  |  |  |
| Respiratory cluster | 91.1 (70.2, 97.2) | 92.9 (73.9, 97.8) | 92.5 (81.5, 97.3) |
| Cancer/osteoarthritis cluster | 85.0 (63.5, 94.8) | 84.5 (62.1, 94.7) | 84.9 (65.2, 94.0) |
| Stroke cluster | 74.5 (68.0, 93.5) | 73.4 (65.9, 93.4) | 72.5 (57.7, 95.3) |
| Mental health cluster | 79.1 (61.7, 96.6) | 76.9 (60.0, 96.4) | 76.7 (73.2, 92.3) |
| Heart cluster | 87.2 (68.2, 96.8) | 88.1 (67.8, 97.7) | 85.7 (66.1, 95.9) |
| Total | 86.2 (68.2, 96.4) | 86.8 (67.9, 96.5) | 86.1 (68.8, 96.4) |
| **Females** |  |  |  |
| Respiratory cluster | 95.5 (79.2, 97.9) | 94.7 (78.3, 97.2) | 96.7 (84.6, 98.7) |
| Thyroid cluster | 94.1 (68.9, 97.9) | 92.8 (69.1, 97.1) | 83.0 (80.3, 91.0) |
| Cancer/osteoarthritis cluster | 94.3 (82.9, 98.1) | 94.6 (84.3, 98.1) | 91.9 (76.7, 96.7) |
| Mental health cluster | 79.6 (62.0, 92.6) | 80.2 (63.9, 93.1) | 78.6 (58.8, 89.9) |
| Heart/stroke cluster | 83.3 (63.4, 96.4) | 82.2 (63.8, 95.3) | 86.3 (65.9, 97.5) |
| Total | 91.7 (72.7, 97.6) | 91.1 (72.7, 97.0) | 88.4 (73.2, 96.2) |

### Table S9: proportion of people with posterior probability < 70%

| Cluster | Posterior probability < 70%, n | Posterior probability >= 70%, n | Proportion of posterior probability < 70%, % |
| --- | --- | --- | --- |
| Males |  |  |  |
| Respiratory | 1265 | 4322 | 22.6 |
| Mental health | 1000 | 1814 | 35.5 |
| Cancer/osteoarthritis | 2009 | 4051 | 33.2 |
| Stroke | 494 | 1138 | 30.3 |
| Heart | 1227 | 2863 | 30 |
| Total | 5995 | 14188 | 29.7 |
| Females |  |  |  |
| Respiratory | 655 | 3461 | 15.9 |
| Mental health | 994 | 2475 | 28.7 |
| Cancer/osteoarthritis | 491 | 2905 | 14.5 |
| Heart/stroke | 575 | 1170 | 33 |
| Thyroid | 796 | 2297 | 25.7 |
| Total | 3511 | 12308 | 22.2 |

### Table S10: Associations (HR (95%CI)) between the disease clusters and all-cause mortality, and mortality of cardiovascular diseases and extrahepatic cancers, hepatocellular carcinoma and liver related diseases in males and females with SLD in sensitivity analysis removing individuals with posterior probability < 70%

|  |  |  | Mortality from | | | | | | |
| --- | --- | --- | --- | --- | --- | --- | --- | --- | --- |
| Clusters | All cause mortality |  | Extrahepatic cancers |  | Cardiovascular disease |  | HCC |  | Liver related diseases |
| Males |  |  |  |  |  |  |  |  |  |
| No MM | Reference |  | Reference |  | Reference |  | Reference |  | Reference |
| MM | 2.03 (1.95, 2.11) |  | 1.47 (1.38, 1.57) |  | 2.76 (2.57, 2.97) |  | 1.63 (1.12, 2.36) |  | 1.79 (1.47, 2.17) |
| Respiratory | 1.56 (1.45, 1.68) |  | 1.23 (1.09, 1.39) |  | 1.72 (1.49, 1.98) |  | 1.23 (0.60, 2.52) |  | 1.41 (0.99, 2.01) |
| Mental health | 1.84 (1.65, 2.05) |  | 1.16 (0.95, 1.43) |  | 2.07 (1.68, 2.55) |  | 2.34 (1.01, 5.44) |  | 2.36 (1.57, 3.54) |
| Cancer/osteoarthritis | 1.86 (1.74, 1.99) |  | 1.94 (1.75, 2.14) |  | 1.46 (1.25, 1.71) |  | 1.33 (0.67, 2.64) |  | 1.77 (1.27, 2.47) |
| Stroke | 2.36 (2.14, 2.61) |  | 1.54 (1.28, 1.85) |  | 3.42 (2.88, 4.07) |  | 1.53 (0.55, 4.24) |  | 1.55 (0.87, 2.76) |
| Heart | 2.85 (2.68, 3.04) |  | 1.31 (1.14, 1.49) |  | 6.05 (5.50, 6.67) |  | 2.24 (1.22, 4.10) |  | 2.11 (1.48, 3.01) |
|  |  |  |  |  |  |  |  |  |  |
| Females |  |  |  |  |  |  |  |  |  |
| No MM | Reference |  | Reference |  | Reference |  | Reference |  | Reference |
| MM | 1.80 (1.70, 1.90) |  | 1.42 (1.30, 1.54) |  | 2.28 (2.02, 2.59) |  | 1.26 (0.53, 2.99) |  | 1.47 (1.10, 1.97) |
| Respiratory | 1.66 (1.50, 1.83) |  | 1.35 (1.16, 1.56) |  | 1.76 (1.40, 2.21) |  | 2.65 (0.88, 8.04) |  | 1.13 (0.64, 1.99) |
| Mental health | 1.50 (1.34, 1.69) |  | 1.18 (0.98, 1.41) |  | 1.75 (1.35, 2.28) |  | NA |  | 1.61 (0.94, 2.75) |
| Cancer/osteoarthritis | 1.94 (1.76, 2.14) |  | 1.95 (1.70, 2.23) |  | 1.84 (1.44, 2.35) |  | 0.85 (0.11, 6.47) |  | 1.59 (0.93, 2.70) |
| Heart/stroke | 3.21 (2.89, 3.57) |  | 1.69 (1.39, 2.06) |  | 6.64 (5.51, 8.01) |  | 2.58 (0.56, 11.89) |  | 2.23 (1.20, 4.15) |
| Thyroid | 1.39 (1.24, 1.55) |  | 1.08 (0.91, 1.28) |  | 1.62 (1.26, 2.09) |  | 0.69 (0.09, 5.20) |  | 1.28 (0.73, 2.27) |

Model was stratified by region and age groups, adjusted for ethnicity, education, Townsend Deprivation Index, physical activity, alcohol intake and smoking. HCC: hepatocellular carcinoma. NA: not applicable. HR (95%CI): hazard ratio (95% confidence interval).
